# A Novel Role of A_2A_R in the Maintenance of Intestinal Barrier Function of Enteric Glia from Hypoxia-Induced Injury by Combining with mGluR5

**DOI:** 10.3389/fphar.2021.633403

**Published:** 2021-05-10

**Authors:** Lihua Sun, Xiang Li, Haidi Guan, Shuaishuai Chen, Xin Fan, Chao Zhou, Hua Yang, Weidong Xiao

**Affiliations:** Department of General Surgery, Xinqiao Hospital, Army Medical University, Chongqing, China

**Keywords:** A_2A_R, intestinal epithelial barrier, mGluR5, enteric glial cells, hypoxia

## Abstract

During acute intestinal ischemia reperfusion (IR) injury, the intestinal epithelial barrier (IEB) function is often disrupted. Enteric glial cells (EGCs) play an important role in maintaining the integrity of IEB functions. However, how EGCs regulate IEB function under IR stimulation is unknown. The present study reveals that the adenosine A_2A_ receptor (A_2A_R) is important for mediating the barrier-modulating roles of EGCs. A_2A_R knockout (KO) experiments revealed more serious intestinal injury in A_2A_R KO mice than in WT mice after IR stimulation. Moreover, A_2A_R expression was significantly increased in WT mice when challenged by IR. To further investigate the role of A_2A_R in IEB, we established an *in vitro* EGC-Caco-2 co-culture system. Hypoxia stimulation was used to mimic the process of *in vivo* IR. Treating EGCs with the CGS21680 A_2A_R agonist attenuated hypoxia-induced intestinal epithelium damage through up-regulating ZO-1 and occludin expression in cocultured Caco-2 monolayers. Furthermore, we showed that A_2A_R and metabotropic glutamate receptor 5 (mGluR5) combine to activate the PKCα-dependent pathway in conditions of hypoxia. This study shows, for the first time, that hypoxia induces A_2A_R-mGluR5 interaction in EGCs to protect IEB function via the PKCα pathway.

## Introduction

Ischemia-reperfusion (IR) injury of the intestine is a fatal syndrome in abdominal surgeries involving aortic aneurysm, small bowel or liver transplantation, cardiopulmonary bypass, strangulated hernias, and neonatal necrotizing entero colitis (Mallick et al., 2004). Acute intestinal IR injury is one of the most important causes of disruption to the intestinal epithelial barrier (IEB), initiates the systemic inflammatory response syndrome, and leads to multiple organ disorders (Vollmar and Menger, 2011; Lu et al., 2012). For these reasons, increasing attention has been focused on the underlying mechanisms of intestinal IR and promising protective strategies.

Traditionally, enteric glial cells (EGCs), the most abundant cell type in the intestinal nervous system, have been proposed to provide trophic and supportive effects for enteric neurons (Aube et al., 2006; Ruhl, 2005). However, accumulating evidence reveals that EGCs also play an important role in the regulation of intestinal epithelial proliferation and the intestinal mucosal defense system (Neunlist et al., 2013; Cheadle et al., 2013). EGCs are responsible for enhanced gut permeability and barrier dysfunction in inflammatory bowel disease (IBD) (Zhang et al., 2010; Cabarrocas et al., 2003; von Boyen and Steinkamp, 2010; Neunlist et al., 2008). In transgenic mice, the conditional deletion of EGCs results in the development of fulminant intestinal inflammation with mucosal barrier breakdown (Bush et al., 1998). Previously, we demonstrated that EGCs enhance IEB functions in response to lipopolysaccharide (LPS) stimulation by inhibiting increased iNOS activity (Xiao et al., 2011). We also found that EGC-released glial-derived neurotrophic factor (GDNF) is closely involved in the IEB protective mechanisms of EGCs in acute IR stimulation (Xiao et al., 2014). However, the precise mechanisms by which EGCs regulate IEB in IR injury have not been elucidated.

The Adenosine A_2A_ receptor (A_2A_R), one of four G protein–coupled adenosine receptors (including A_1_R, A_2A_R, A_2B_R, and A_3_R), binds adenosine and induces activation of adenylate cyclase, promoting cAMP synthesis and producing corresponding biological effects (Welihinda et al., 2016). A_2A_R is involved in the regulation of several physiological functions, including in the gastrointestinal system (Fornai et al., 2009). A_2A_R has diverse and important roles in the intestine, including gut motor functions, acetylcholine release, cholinergic contraction modulation, and enteric nervous system regulation (Duarte-Araújo et al., 2004; Antonioli et al., 2006; Fornai et al., 2009; Schriemer et al., 2016). DucoSchriemer et al. revealed that A_2A_R is a key regulator of terminal neuronal differentiation in GDNF-treated enteric neural crest cells (ENCCs) (Schriemer et al., 2016). However, there is relatively little information about the role of A_2A_R in EGCs. Therefore, this study was designed to investigate the role of A_2A_R in EGC-mediated IEB regulation.

## Materials and Methods

### Cell Culture and Co-Culture

Rat EGC/PK060399egfr (CRL-2690™) and human intestinal epithelial cells Caco-2 (HTB-37™) were obtained from the American Type Culture Collection. EGC/PK060399egfr and Caco-2 cells were grown in high glucose DMEM and MEM, respectively, supplemented with 10% FCS, 2 mM l-glutamine, and 100 U/100 μg/ml penicillin–streptomycin. Cells were incubated in a 5% CO_2_ humidified incubator at 37°C. The EGC-Caco-2 co-culture system was established as described previously by our laboratory (Xiao et al., 2014). Caco-2 cells were seeded on Millicell®filters (0.4 μm pore diameter; Millipore; Billerica, MA) at a density of 5 × 10^4^ cells/cm^2^ for up to 4–5 days. EGCs were seeded at an equal density in 6 or 24 well tissue culture plates to avoid any possible direct cell contact with Caco-2 cells. During the co-culture period, half of the culture medium in the apical and basal compartments was changed daily.

### Mice

Global A_2A_R homozygous KO mice (A_2A_R^−/−^ mice) with C57BL/6J background were provided by Dr. Yuanguo Zhou (Research Institute of Surgery, Daping Hospital, Army Medical University, Chongqing, China). Specific pathogen-free wild-type (WT) C57BL/6J mice were purchased from the Laboratory Animal Center of the Army Medical University. All mice were housed and maintained in laminar flow cabinets under specific pathogen-free conditions.

### In Vitro Hypoxia Experiments

For hypoxia experiments, cells were subjected to hypoxia in a CO_2_ incubator (Forma® Series II Water Jacketed CO_2_ Incubators; Thermo Scientific) with 94% nitrogen, 5% CO_2_, and 1% oxygen and incubated at 37°C for 6 h. Re-oxygenation was initiated by replacing the media and exposing the cell monolayers to 37°C plus 5% CO_2_for 1 h. Control cells were maintained at 37°C in an atmosphere with 5% CO_2_.

### Intestinal Ischemia/Reperfusion Model

Male mice (8–10 weeks old) were fasted for 12 h and were free to drink water prior to surgery. The animals were intraperitoneally injected with 40 mg/kg of pentobarbital anesthesia and an aseptic laparotomies dioventral line was placed. The following specific surgical procedures were performed as previously described (Xiao et al., 2014).

### Western Blot Analysis

Cells and tissues were lyzed in cold RIPA buffer for 30 min and centrifuged at 13,000× *g* for 30 min at 4°C. Protein concentration was determined using a BCA assay reagent (Beyotime). The primary antibodies used were rabbit anti-ZO-1 (1:800), rabbit anti-Occludin (1:1000), mouse anti-A_2A_R (1:500), rabbit anti-PKCα (1:1000), rabbit anti-Na+/K+ ATPase (1:1000), and rabbit anti-GAPDH (1:1,000). Protein expression was measured in optical density units and normalized to GAPDH expression.

### Immunofluorescence Staining

The small intestine tissues were embedded with OCT compound (Tissue-Tek, Sakura Finetek, Torrance, CA, United States). Consecutive frozen sections (5 μm in thickness) were obtained and fixed in 4% paraformaldehyde for 20 min at room temperature. After 30 min pre-incubation with a blocking solution containing 5% bovine serum albumin, sections were incubated overnight at 4°C with primary antibody against GFAP (Abcam), A_2A_R (Abcam), or ZO-1 (Abcam). After washing in PBS, sections were incubated with fluorescence-conjugated secondary antibodies at 37°C for 1 h. After washing in PBS, sections were incubated with DAPI nuclear stain solution for 5 min. All images were obtained using a TCS-SP5 confocal microscope (Leica, Germany).

### Coimmunoprecipitation

Cells were harvested and lyzed in standard immunoprecipitation (IP) buffer containing either 1% 3-[(3-cholamidopropyl) dimethylammonio] propanesulfonic acid (Chaps) or 1% Triton X-100 (for ERAD substrates), or 2% digitonin (for ERAD machinery) for 1 h on ice. Cells were centrifuged at 16,000 × *g* for 10 min, and the supernatant was used for immunoprecipitation experiments. Co-IP was performed using protein A-agarose beads (EMD Millipore) with anti-A_2A_R (Santa Cruz Biotechnology, Inc.), anti-mGluR5 antibodies (Cell signaling), or anti-D2R antibodies (Santa Cruz Biotechnology, Inc.) following the usual method.

### Transepithelial Electrical Resistance (TER) and Permeability Measurements

The TER of cells was determined via a Millipore electric resistance system (ERS-2; Millipore). Caco-2 cell monolayers were grown in Millicell inserts (0.33 cm^2^ area, 0.4 μm pore diameter, and 6.5 mm diameter) and the culture medium was replaced before TER measurement. To calculate the actual resistance of the cell monolayer, the mean resistance of filters without cells was subtracted from the monolayer measurement, and the difference between the filter and monolayer areas was corrected.

The intestinal mucosa TER was measured by Ussing chambers (Physiologic Instruments, San Diego, CA). The excised intestinal tissues were bathed in 5 ml Krebs buffer (110.0 mM NaCl, 3.0 mMCaCl_2_, 5.5 mM KCl, 1.4 mM KH_2_PO_4_, 29.0 mM NaHCO_3_, and 1.2 mM MgCl_2_, pH 7.4)on both the mucosal and serosal sides. The TER was measured as previously described (Liu et al., 2018).

### Statistical Analysis

All experimental data are shown as the Mean ± SD. Statistical significance was determined by unpaired two-tailed Student *t* test analysis using GraphPad Prism version 7.0 software (San Diego, CA). If not otherwise stated, all experiments included three independent replications in triplicate. *p* < 0.05 was considered statistically significant.

## Results

### Acute IR Treatment Significantly Activated A_2A_R Expression in Intestinal Mucosa EGC

**FIGURE 1:**
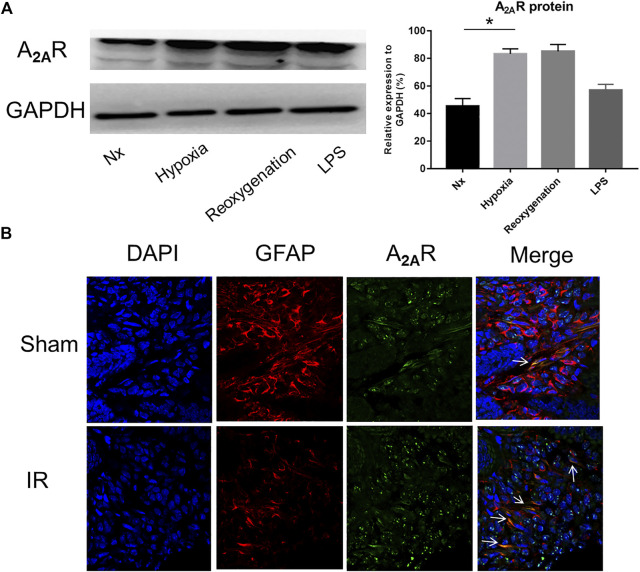
Expression of A_2A_R in enteric glial cells (EGCs) under hypoxia stimulation. **(A)** Western blot analysis shows a significant increase of A_2A_R protein level in cultured EGC cells after hypoxia and reoxygenation treatment. GAPDH was used as a standard for cellular protein input. **(B)** Immunofluorescence was used to detect A_2A_R expression in the small intestine under IR treatment. Red and green signals represent GFAP and A_2A_R, respectively. Results are expressed as mean ± SD (*n* = 4–6 mice/group) (*p < 0.05).

To study the effect of A_2A_R on EGC, we first examined the expression of A_2A_R in different pathological conditions. Lipopolysaccharide (LPS) and hypoxia treatments were used to stimulate EGCs *in vitro*. As shown in Figure 1A, A_2A_R expression dramatically increased in EGCs following LPS and hypoxia stimulation, with the effect of hypoxia being obvious than that of LPS. Therefore, hypoxia treatment was used to study the effect of A_2A_R. As demonstrated previously, GFAP is a specific marker of activated glial cells (Xiao et al., 2014). A_2A_R and GFAP immunofluorescent staining colocalization were used to observe changes in A_2A_R expression in EGCs. Acute IR-treated mice showed a moderate decrease in GFAP-positive intestinal EGCs compared to sham-treated mice (Figure 1B). However, the A_2A_R levels increased significantly in GFAP-positive EGCs after IR treatment. Together, these results indicate that hypoxia stimulation can activate the A_2A_R-mediated signaling pathway in mucosal EGCs in the intestine.

### Activation of A_2A_R in EGCs Efficiently Prevents Barrier Dysfunction of Caco-2 Monolayers Under Hypoxia Stimulation

**FIGURE 2:**
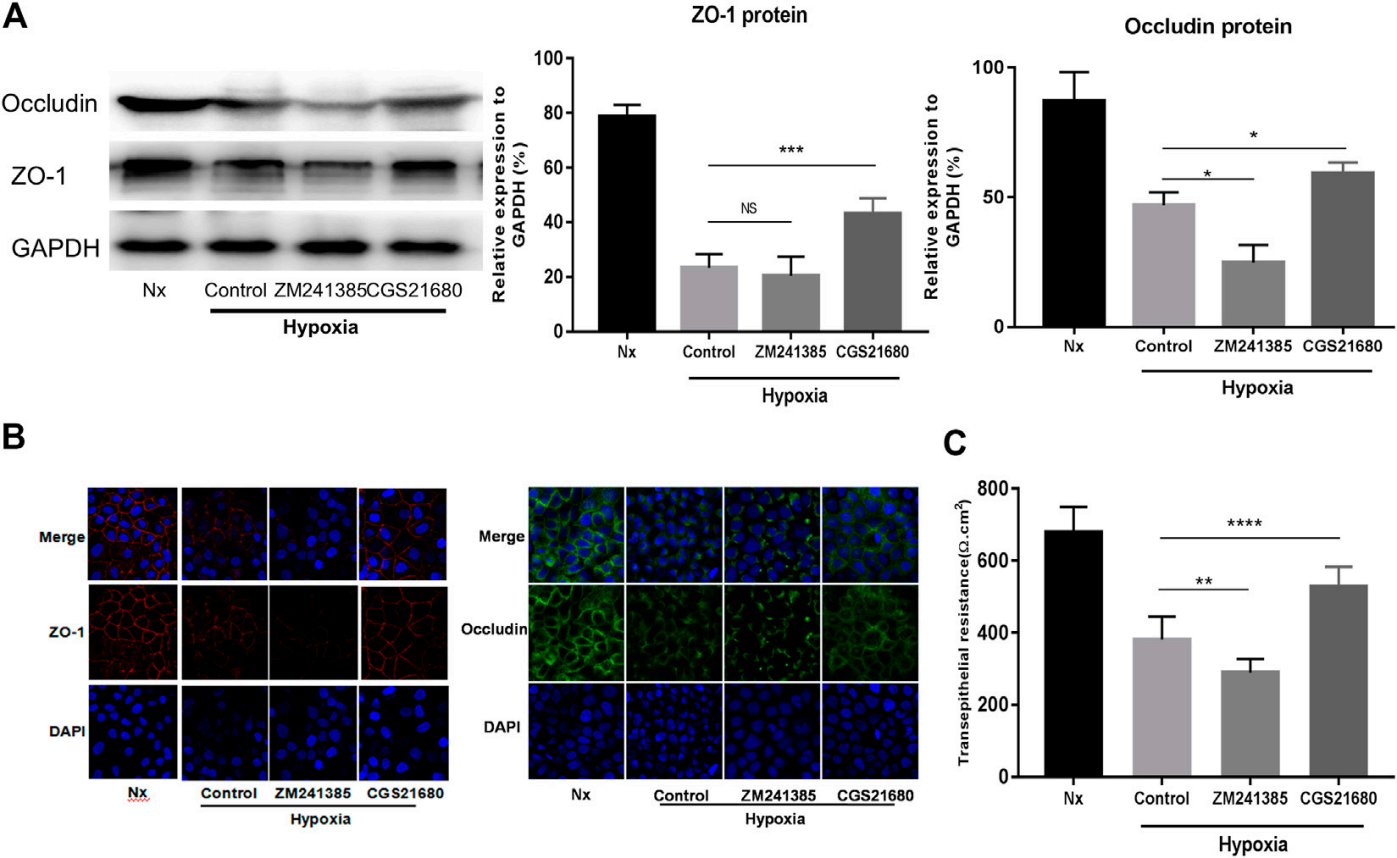
Activation of A_2A_R in enteric glial cells (EGCs) prevents Caco-2 monolayer barrier dysfunction under hypoxia stimulation. **(A–C)** EGCs were co-cultured with Caco-2 monolayers for 24 h and then treated with ZM241385 (1 μM) and CGS21680 (100 nM) for 6 h respectively under hypoxia conditions. **(A)** Western blot and **(B)** immunofluorescence analyses were used to detect ZO-1 and occludin expression. **(C)** The transepithelial electrical resistance (TER) of Caco-2 monolayers was determined to evaluate intestinal epithelial barrier (IEB) function. Results are expressed as mean ± SD. (**p < 0.01; ***p < 0.001; ****p < 0.0001; NS, not significant.)

To further explore the role of A_2A_R in IEB modulation under acute hypoxia stimulation, we used an A_2A_R agonist and inhibitor separately in an *in vitro* EGC-Caco-2 co-culture system. The tight junctions (TJs) are primary determinants of IEB function (Lee, 2015). As shown in Figure 2A, western blot analysis revealed an almost 50% drop in ZO-1 and occludin expression in the hypoxia group and a similar reduction on ZO-1 and occludin expression in the A_2A_R inhibitor ZM241385 group. However, the A_2A_R agonist CGS21680 significantly prevented hypoxia-induced TJs destruction. Further study with immunocytochemistry (ICC) confirmed the western blot results (Figure 2B). Additionally, TER measurement analysis produced similar results. CGS21680 pretreatment effectively blocked TER decrease under hypoxia stimulation when compared to the ZM241385 pretreatment group (528.6 ± 18.11 Ω cm^2^ vs. 289.6 ± 12.63 Ω cm^2^ for the CGS21680 and ZM241385 pretreatment groups, respectively) (Figure 2C). Together, these results showed that A_2A_R plays a protective role in the EGC barrier-protecting effect on the IEC monolayer under hypoxia stimulation.

### A_2A_R Deficiency Aggravates IR-Induced IEB Injury

**FIGURE 3:**
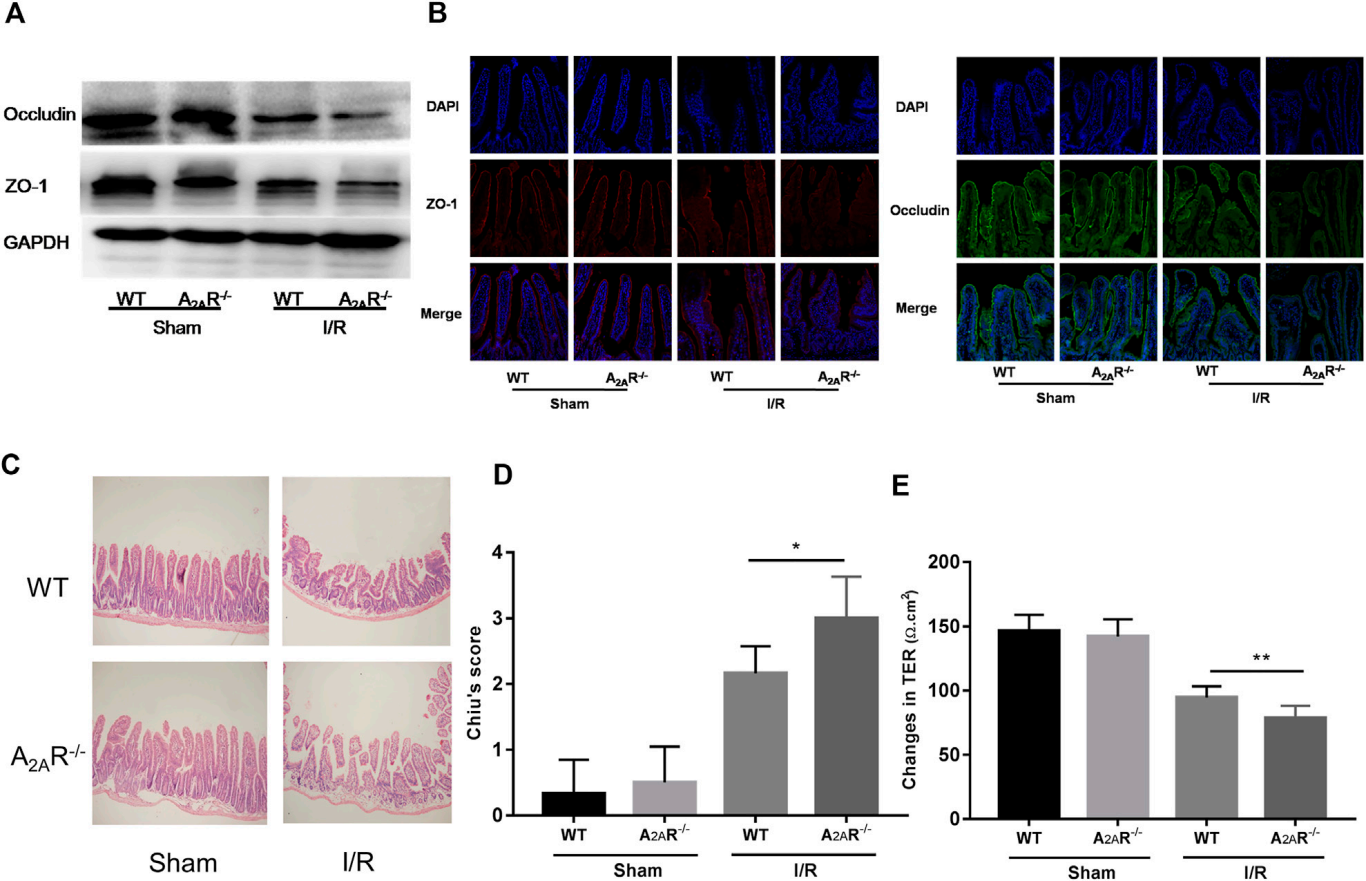
A_2A_R deficiency aggravates IR-induced intestinal epithelial barrier (IEB) injury. IEB function was assessed in IR-treated WT and A_2A_R^−/−^ mice by **(A)** ZO-1 and occludin protein expression measured by western blot **(B)**, ZO-1 and occludin protein expression measured by immunofluorescence, and **(E)** the small intestine TER value. The degree of intestinal damage was assessed in IR-treated WT and A_2A_R^−/−^ mice by **(C)** hematoxylin and eosin staining, and **(D)** Chiu’s Score. Results are expressed as mean ± SD (*n* = 4–6 mice/group. *p < 0.05; **p < 0.01).

To further confirm the role of A_2A_R in the barrier protective of EGCs during hypoxia stimulation, A_2A_R KO mice were treated with IR. Western blot analysis of ZO-1 and occludin expression was assessed in the small intestine (Figure 3A). Acute IR treatment led to a substantial decrease in ZO-1 and occludin expression in A_2A_R KO mice compared to WT mice. Immunofluorescence analysis also revealed a similar reduction in ZO-1 and occludin expression in the intestinal mucosa after IR stimulation (Figure 3B). The functional impact of A_2A_R knockdown on tight junctions in the small intestine was further evaluated by determining the TER value using Ussing chambers. As shown in Figure 3E, intestinal I/R caused a marked TER decrease in A_2A_R KO mice (78.63 ± 3.407 Ω cm^2^) compared with WT mice (94.5 ± 3.151 Ω cm^2^). Histological examination of intestinal tissues revealed that IR-treated A_2A_R KO mice showed more increased intestinal villus fracturing and epithelial removal than did WT mice (Figures 3C,D). Together, these results strongly suggest that A_2A_R has a significant protective effect in IR-induced IEB injury.

### Hypoxia Induces the Interaction between A_2A_R and mGluR5

**FIGURE 4:**
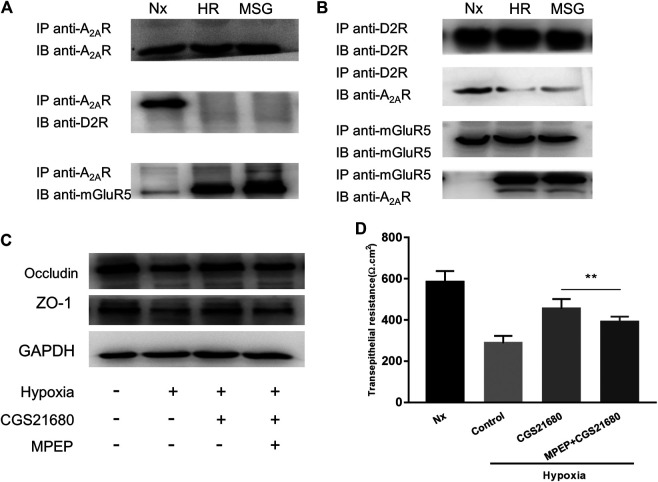
The protective effect of A_2A_R on the intestinal epithelial barrier (IEB) is mediated by combining with mGluR5. **(A)** Co-IP of A_2A_R and D2R or mGluR5 in enteric glial cells (EGCs) using an anti-A_2A_R precipitating antibody. **(B)** Interactions between A_2A_R and D2R or mGluR5 in EGCs were confirmed by Co-IP using anti- D2R or anti-mGluR5 precipitating antibodies. **(C**,**D)** EGCs were co-cultured with Caco-2 monolayers for 24 h and then treated with MPEP (100 μM) and CGS21680 (100 nM) for 6 h respectively under hypoxia conditions. **(C)** Western blot and **(D)** TER analysis were used to detect the function of IEB. Results are expressed as mean ± SD. (**p < 0.01).

There are synergistic interactions between A_2A_R, mGluR5, and the dopamine D2 receptor (D2R) in central nervous system (CNS) related diseases (Ferraro et al., 2012; Fernández-Dueñas et al., 2013). However, whether this relationship exists in the enteric nervous system has yet to be determined. It has been suggested that in micro glial cells, A_2A_R combines with D2R in low glutamate concentration and combines with mGluR5 in high glutamate concentration (Dai et al., 2010; Beggiato et al., 2016). Therefore, we used high glutamate concentrations as a positive control. We studied the relationship among them under hypoxia in EGCs. When exploring the effect of oxygen concentration, the band corresponding to D2R was coimmunoprecipitated by anti-A_2A_R antibodies, and an A_2A_R band was coimmunoprecipitated by anti-D2R antibodies. Together, these results indicate that A_2A_R and D2R interact in normoxic but not in hypoxic conditions (Figure 4A). However, an opposite relationship was observed between A_2A_R and mGluR5. As shown in Figure 4B, the band corresponding to mGluR5 was coimmunoprecipitated by anti-A_2A_R antibodies, and an A_2A_R band was also coimmunoprecipitated by anti-mGluR5 antibodies. These results indicate that A_2A_R and D2R interact in hypoxia but not in normoxia, demonstrating that A_2A_R interacts with D2R under normoxic conditions and interacts with mGluR5 under hypoxic conditions.

### mGluR5 Inhibition Attenuates the Protective Effect of A_2A_R on the IEB from Hypoxia Induced Damage

To confirm that A_2A_R regulates the IEB via a mGluR5-dependent pathway, we tested the responsiveness of the IEB to 100 μM of the MPEP selective mGluR5 antagonist under hypoxia. Western blot analysis of ZO-1 and occludin expression revealed that treatment with MPEP significantly inhibited CGS21680-mediated activation of ZO-1 and occludin expression after hypoxia induced damage (Figure 4C). Similar results were observed in TER measurements: CGS21680 pretreatment effectively prevented the decrease of TER from hypoxia stimulation, while, MPEP blocked CGS21680-induced potentiation (454.3 ± 15.7 Ω cm^2^ vs. 391 ± 8.441 Ω cm^2^) (Figure 4D). Together, these results indicate that the protective effect of A_2A_Ron the IEB after hypoxia stimulation is dependent on mGluR5.

### The PKCα Signaling Pathway Is Required for the Protective Function of A_2A_R on IEB

**FIGURE 5:**
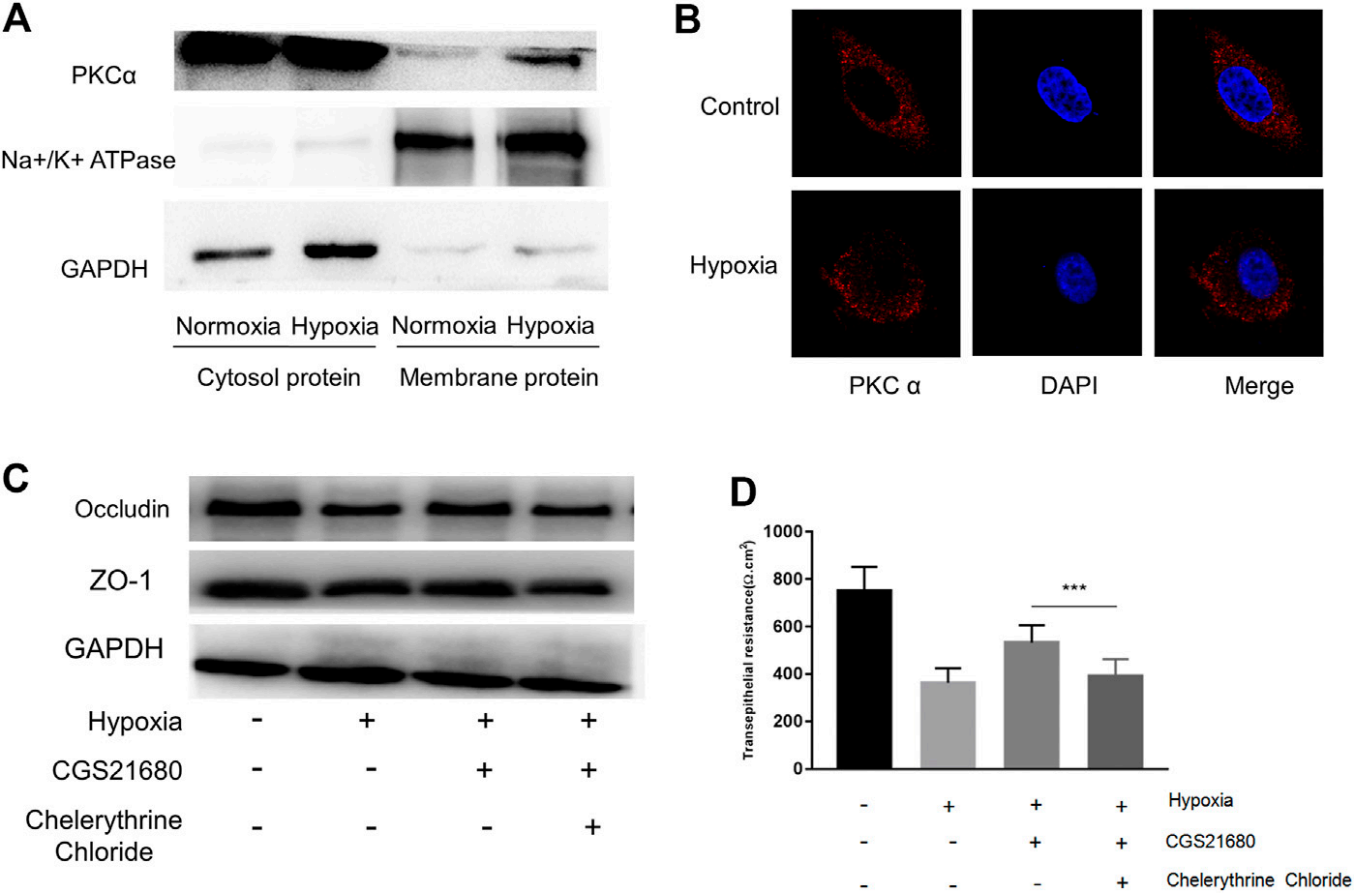
The protective effect of A_2A_R on IEB is PKCα dependent. PKCα expression was assessed in different enteric glial cells (EGCs) fractions. **(A)** Membrane and cytosol proteins were respectively extracted to detect PKCα change under hypoxia. **(B)** Immunofluorescence was used to visualize PKCα distribution under hypoxia. **(C**,**D)** EGCs were co-cultured with Caco-2 monolayers for 24 h, then treated with chelerythrine chloride (10 μM) and CGS21680 (100 nM) for 6 h under hypoxic conditions. **(C)** Western blot and **(D)** TER analysis were used to detect the function of IEB. Results are expressed as mean ± SD. (***p < 0.001).

The PKCα signaling pathway is associated with an A_2A_R–mGluR5 interaction-associated proinflammatory effect (Dai et al., 2013; Li et al., 2017). Therefore, we next tested whether PKCα is required for A_2A_R-mediated IEB protection. PKC family isoforms can translocate to multiple subcellular localizations in response to hypoxia in different cell lines (Yu et al., 2015). Consistent with this, western blots showed increased PKCα expression in the membrane after hypoxia stimulation in EGCs (Figure 5A). These results were confirmed by immunocytochemistry analysis showing that PKCα translocates from the cytoplasm to the cell membrane in EGCs after hypoxia stimulation (Figure 5B). We then used PKCα inhibitor, chelerythrine chloride, to observe whether the protective effect of CGS21680 on IEB was affected. As shown in Figure 5C, chelerythrine chloride significantly reduced ZO-1 and occludin protein levels in CGS21680-pretreated EGCs. As expected, chelerythrine chloride pretreatment also blocked CGS21680-promoted TER (533 ± 24.2 Ω cm^2^ vs. 390.9 ± 24.14 Ω cm^2^) (Figure 5D). Together, these results suggest that A_2A_R exerts its protective effects on IEB via the PKCα signaling pathway.

## Discussion

Our research has previously demonstrated that EGCs enhance IEB functions under acute intestinal injury (Xiao et al., 2014). In this context, our data provide the first evidence that EGCs protect IEB by activating A_2A_R. The A_2A_R agonist significantly improved the barrier functions of Caco-2 monolayers following exposure to HR stimulation. Moreover, in A_2A_R KO mice, intestinal tissue damage was accelerated, including the structural and mucosal barrier defects, following intestinal I/R. We found that A_2A_R combines with mGluR5 under hypoxic conditions to exert a protective effect on IEB. This data also shows that A_2A_R and mGluR5 combine to activate the PKCα-dependent signaling pathway. Together, these results show that A_2A_R plays a critical role in the barrier protective mechanism of EGCs under acute intestinal hypoxia stimulation.

EGCs are involved in the regulation of IEB function. However, the precise mechanisms by which EGCs function in the regulation of IEB remain unclear. Increasing evidence indicates that EGCs and astrocytes share morphological features and electrophysiological properties and express similar proteins, including GFAP and S100β, leading to the idea that EGCs might share many features of the central nervous system astrocytes (Le Berre-Scoul et al., 2017). The similarities between EGCs and astrocytes indicate that these two glial cell types may regulate barrier functions through common molecular mechanisms (Jiang et al., 2005; Xiao et al., 2014). It is reported that primary cell cultures of either astrocytes or enteric glia can induce barrier properties across endothelia and epithelia (Savidge et al., 2007). Jiang Set al. previously reported that implantation of enteric glia accelerates normal spinal cord vasculature repair processes at the site of injury and promotes functional blood-brain barrier (BBB) induction (Jiang et al., 2005). Additionally, glia promote blood-brain barrier-like properties in peripheral sites including blood-ocular barriers in the eye, the perineurium of peripheral nerves, and the blood myenteric plexus barrier in the gut (Gershon and Bursztajn, 1978; Savidge et al., 2007). Our previous LPS and hypoxia reperfusion stimulation studies showed that EGCs can effectively alleviate IEB damage (Xiao et al., 2011; Xiao et al., 2014). Therefore, we explored the mechanism by which EGCs protect the IEB.

A_2A_R activation is closely related to a variety of neurological diseases and is an important component of the adenosine signaling pathway (Stone et al., 2009). Recently, many studies have suggested that A_2A_R also plays an important protective role in enteritis (Warren et al., 2012; Antonioli et al., 2018). However, there is relatively little information about the role of A_2A_R in intestinal IR damage. A_2A_R inactivation can prevent IR by regulating the inflammatory response and excitotoxic cascades in the brain, kidney, lung, and blood vessels (Vincent and Okusa, 2015; Li et al., 2013; Mohamed et al., 2012; Mohamed et al., 2016; Gui et al., 2009; Cunha, 2005). Due to the similarities between the brain and the intestine, we speculate that A_2A_R may also have protective effects on intestinal IR damage. Our data shows that IEB damage is accelerated in A_2A_R KO mice. However, because there are no mice with selective inactivation of EGCs-derived A_2A_R, we cannot comprehensively show that EGCs protect IEB via the A_2A_R pathway. We exposed an *in vitro* EGC-Caco-2 co-culture system to hypoxia treatment to detect the role of A_2A_R in EGCs. Our results show that activation of A_2A_R in EGCs prevents damage to the IEB during hypoxia.

To clarify the mechanism by which A_2A_R influences IEB functions under acute intestinal epithelium hypoxia injury, we explored how A_2A_R works in the brain. Functional A_2A_R-mGluR5 heteromeric complexes have been reported in the central nervous system (Beggiato et al., 2016; Temido-Ferreira et al., 2018). Beggiato et al. found that A_2A_R and mGlu5R interact synergistically to modulate D2R-mediated control of striatopallidal GABA neurons (Beggiato et al., 2016). Additionally, Dai et al. reported that A_2A_R-mGluR5 interplay is critical for the proinflammatory effect in bone marrow-derived cells (BMDCs) after acute lung injury (Dai et al., 2013). Consistent with our expectation, we observed that A_2A_R combined with mGluR5 in EGCs suffering from hypoxia.

mGluR5 is a G-protein-coupled receptor that exerts its physiological roles through intracellular chemical-messenger signaling cascades (Power et al., 2016). In general, mGluR5 represents a promising target for studying neuro-protective agents of potential application in neurodegenerative diseases (Li et al., 2017). However, little data exists supporting the function of mGluR5 in the intestine, especially in relation to its role in IEB regulation. In the intestinal mucosa, mGluR5 is only observed in EGCs (Nasser et al., 2007). EGCs are involved in the occurrence of inflammatory bowel disease through c-Fos and ERK1/2 phosphorylation induced by mGluR5 (Nasser et al., 2007). In the present study, we demonstrated that mGluR5 plays a key role in the protection of IEB by A_2A_R. The proinflammatory effect of mGluR5 is not mediated by PKC signaling, but instead uses the PKA pathway (Dai et al., 2013). Giaroni et al. reported that the PKCα antagonist significantly inhibits intestinal mucosal injury induced by IR (Giaroni et al., 2011). These studies provide further support for our results that A_2A_R protects the IEB by a PKCα dependent pathway.

**FIGURE 6:**
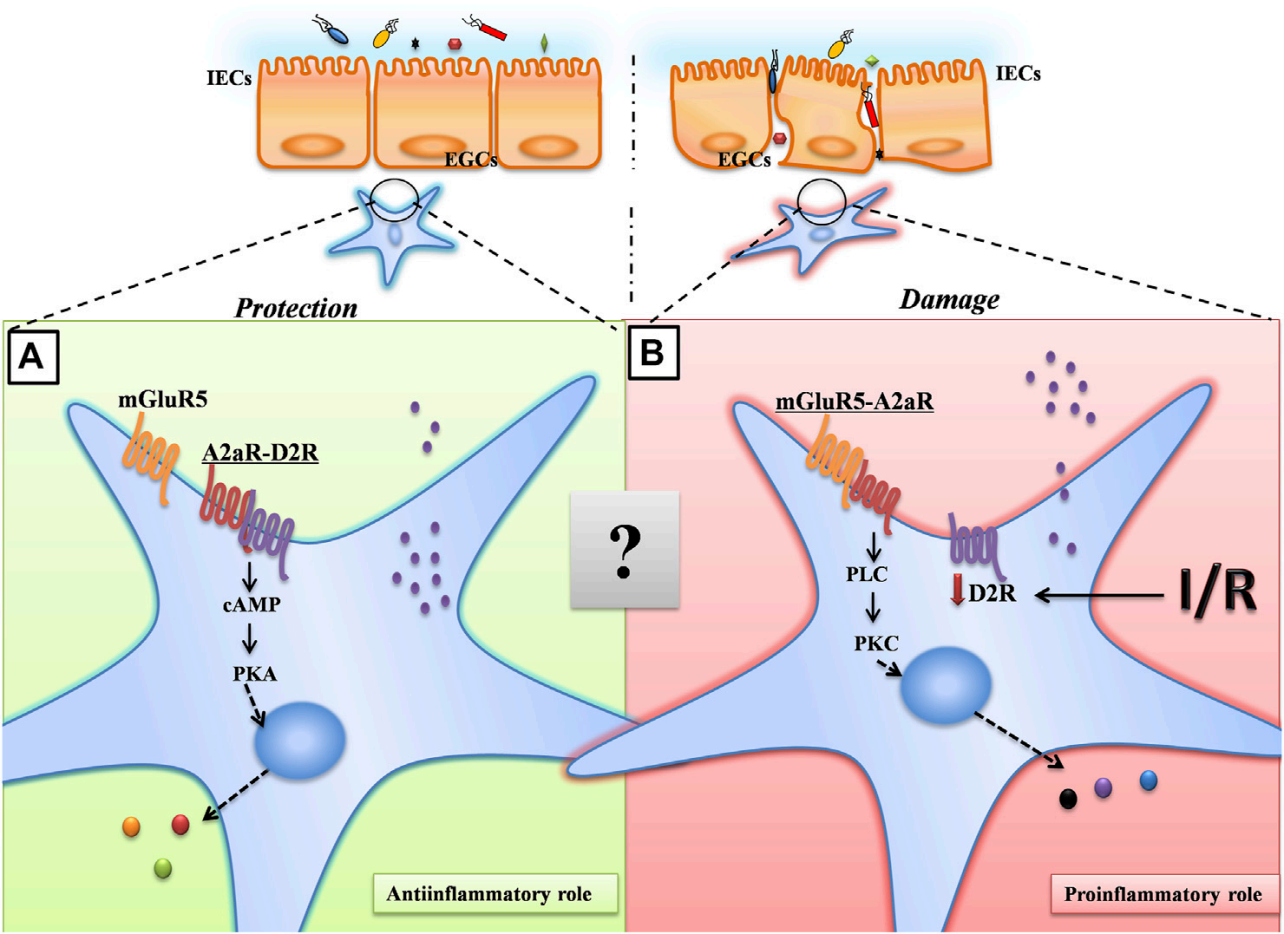
A proposed model for A_2A_R in the maintenance of intestinal barrier function. Under normoxic conditions, A_2A_R combines with D2R to maintain the normal physiological activities of EGCs through the PKA signaling pathway. Under hypoxic conditions, A_2A_R combines with mGluR5 to protect IEB function via the PKCα pathway in EGCs.

Taken together, our results suggest a model for A_2A_R in the maintenance of intestinal barrier function. Upon intestinal hypoxia injury, A_2A_R combines with mGluR5 to protect IEB function via the PKCα pathway in EGCs (Figure 6). Although there are complex interactions between A_2A_R and mGluR5 that remain to be fully understood, our findings are important for a better understanding of the role of EGCs in regulating IEB. Additionally, these findings offer new insight into the clinical use of A_2A_R modulators for IR-induced intestinal injury.

## Data Availability

The raw data supporting the conclusions of this article will be made available by the authors, without undue reservation.
